# Extra-pancreatic invasion induces lipolytic and fibrotic changes in the adipose microenvironment, with released fatty acids enhancing the invasiveness of pancreatic cancer cells

**DOI:** 10.18632/oncotarget.15430

**Published:** 2017-02-17

**Authors:** Takashi Okumura, Kenoki Ohuchida, Masafumi Sada, Toshiya Abe, Sho Endo, Kazuhiro Koikawa, Chika Iwamoto, Daisuke Miura, Yusuke Mizuuchi, Taiki Moriyama, Kohei Nakata, Yoshihiro Miyasaka, Tatsuya Manabe, Takao Ohtsuka, Eishi Nagai, Kazuhiro Mizumoto, Yoshinao Oda, Makoto Hashizume, Masafumi Nakamura

**Affiliations:** ^1^ Department of Surgery and Oncology, Graduate School of Medical Sciences, Kyushu University, Fukuoka, Japan; ^2^ Department of Advanced Medical Initiatives, Graduate School of Medical Sciences, Kyushu University, Fukuoka, Japan; ^3^ Innovation Center for Medical Redox Navigation, Kyushu University, Fukuoka, Japan; ^4^ Department of Anatomic Pathology, Graduate School of Medical Sciences, Kyushu University, Fukuoka, Japan; ^5^ Kyushu University Hospital Cancer Center, Fukuoka, Japan

**Keywords:** pancreatic cancer, extra-pancreatic invasion, adipose microenvironment, lipolysis, fatty acids

## Abstract

Pancreatic cancer progression involves components of the tumor microenvironment, including stellate cells, immune cells, endothelial cells, and the extracellular matrix. Although peripancreatic fat is the main stromal component involved in extra-pancreatic invasion, its roles in local invasion and metastasis of pancreatic cancer remain unclear. This study investigated the role of adipose tissue in pancreatic cancer progression using genetically engineered mice (*Pdx1-Cre; LSL-Kras^G12D^; Trp53^R172H/+^*) and an *in vitro* model of organotypic fat invasion. Mice fed a high fat diet had significantly larger primary pancreatic tumors and a significantly higher rate of distant organ metastasis than mice fed a standard diet. In the organotypic fat invasion model, pancreatic cancer cell clusters were smaller and more elongated in shape and showed increased fibrosis. Adipose tissue-derived conditioned medium enhanced pancreatic cancer cell invasiveness and gemcitabine resistance, as well as inducing morphologic changes in cancer cells and increasing the numbers of lipid droplets in their cytoplasm. The concentrations of oleic, palmitoleic, and linoleic acids were higher in adipose tissue-derived conditioned medium than in normal medium, with these fatty acids significantly enhancing the migration of cancer cells. Mature adipocytes were smaller and the concentration of fatty acids in the medium higher when these cells were co-cultured with cancer cells. These findings indicate that lipolytic and fibrotic changes in peripancreatic adipose tissue enhance local invasiveness and metastasis via adipocyte-released fatty acids. Inhibition of fatty acid uptake by cancer cells may be a novel therapy targeting interactions between cancer and stromal cells.

## INTRODUCTION

Pancreatic cancer (PDAC) is the fourth leading cause of cancer-related deaths worldwide, with a 5-year survival rate of only 6% [1]. Most patients are inoperable at initial diagnosis because of locally advanced disease or distant metastasis [2]. Determining the mechanisms underlying pancreatic cancer cell invasion and metastasis and establishing new therapeutic strategies are therefore urgently needed. One important pathological feature of pancreatic cancer is the abundance of stromal components, or desmoplasia. These components, which include stellate cells, immune cells, endothelial cells and extracellular matrices, have been shown to affect the proliferation and invasiveness of cancer cells and their resistance to therapeutic agents [2, 3]. Several agents targeting these stromal components have been developed [4–7] and tested in clinical trials [8–11], but none has been shown effective. These ineffective outcomes may have been due, at least in part, to the performance of these investigations *in vitro* or in transplantation models using immunodeficient animals, models that may not accurately reflect the highly complex tumor microenvironment *in vivo*. Thus, it is important to establish appropriate experimental models reflecting human PDAC and focus on the novel biological significance of stromal components.

Recent studies of the adipose tumor microenvironment have reported that interactions between cancer cells and adipocytes affect the growth of breast, ovarian, and prostate cancers. Breast cancers, for example, are surrounded by dense adipose tissue, with recruited adipocytes called cancer associated-adipocytes [12]. Moreover, fibroblastic cells derived from these adipocytes contribute to desmoplasia [13]. In ovarian cancer, lipids from omental adipocytes are taken up by cancer cells and consumed to provide energy for rapid tumor growth [14]. In prostate cancer, CCR3/CCL7 from adipocytes promotes the migration of cancer cells [15]. Taken together, these findings indicate that morphological changes in adipose tissue in the tumor microenvironment can affect cancer progression.

The pancreas is a retroperitoneal organ surrounded by adipose tissue, with the latter being a major stromal component in the extra-pancreatic invasion of PDAC. Epidemiological investigations have shown that obesity is a risk factor for pancreatic cancer [16, 17]. In addition, adipose rich stroma was induced in mice with a K-ras mutation deficient in pigment epithelium-derived factor (PEDF), leading to the development of invasive pancreatic carcinoma [18]. Similarly, mice with a Kras mutation fed a high fat diet showed increases in the numbers of precancerous lesions and invasive carcinomas [19–21]. In humans, peripancreatic fat invasion was associated with poor prognosis in patients with PDAC [22]. Taken together, these results indicate that peripancreatic adipose tissue affects the initiation of PDAC. However, little is known about the biological and molecular mechanisms by which interactions between cancer and adipose tissue affect the local invasion and metastasis of PDAC.

This study was designed to evaluate the interaction between pancreatic cancer cells and adipose tissue and to clarify the adipocyte-associated mechanisms of extra-pancreatic invasion and metastasis. An *in vivo* model, consisting of *Pdx1-Cre; LSL-Kras^G12D^; Trp53^R172H/+^* mice fed a high fat diet, and an *in vitro* model of organotypic fat invasion were therefore tested. In addition, interactions between cancer and adipose tissue, focusing on lipid droplets in cancer cells, fatty acid uptake, and lipolysis, were analyzed.

## RESULTS

### Visceral fat induced by a high fat diet enhances primary tumor growth and distant metastasis in KPC mice

**Figure 1:**
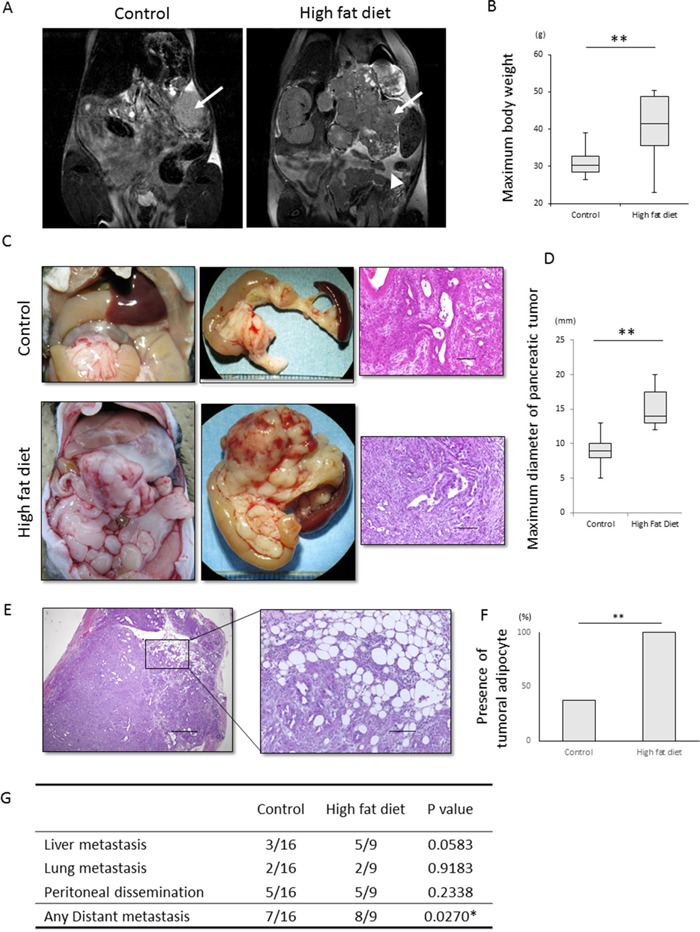
Effect of a high fat diet on the macroscopic appearance and histology of KPC tumors **(A, B)** Effect of high fat diet on visceral fat and body weight. **(A)** Representative T2-weighted MRI images of KPC mice fed a normal diet and a high fat diet. Arrows indicate primary pancreatic tumors. The arrow head in the image of the mouse fed a high fat diet indicates visceral fat around the pancreas. **(B)** Maximum body weight was significantly higher in mice fed a high fat diet (n=9) than a normal diet (n=16). **p<0.001. **(C)** Representative macroscopic and histological images of KPC mice fed a normal diet and a high fat diet. Samples were stained with hematoxylin-eosin. Scale bar, 100 μm. **(D)** Effect of high fat diet on tumor diameter. The maximum diameter of pancreatic tumors was significantly higher in mice fed a high fat diet (n=8) than a normal diet (n=13). **p<0.001. **(E, F)** Effects of high fat diet on tumoral adipocytes. **(E)** Representative image of tumoral adipocytes stained with hematoxylin and eosin. Scale bar. 100 μm. **(F)** Tumoral adipocytes were significantly more frequent in mice fed a high fat (n=8) than a normal (n=13) diet. **p<0.001. **(G)** Effects of high fat diet on distant metastasis and organ specificity. Ingestion of a high fat diet increased the number of distant metastases, but had no effect on organ specificity. *p<0.05.

To assess the effects of peripancreatic fat on invasion and metastasis of PDAC, *Pdx1-Cre; LSL-Kras^G12D^; Trp53^R172H/+^* mice, hereafter called KPC mice, were fed a high fat or normal diet (Supplementry Figure 1A). Body weight and visceral fat were significantly higher in the high fat diet group (p<0.001 each, Figure 1A, 1B, Supplementry Figure 1B, 1C). The maximum diameter of primary pancreatic tumors was significantly greater in the high fat than in the normal diet group (p<0.001, Figure 1C, 1D). Moreover, tumors in the high fat diet group frequently invaded surrounding organs, such as the stomach and small intestine, although the degree of differentiation was similar in the high fat and normal diet groups (Supplementry Figure 1D). The percentages of proliferating cell nuclear antigen (PCNA)-positive cells in pancreatic tumors were similar in the two groups (Supplementry Figure 1E, 1F). Intratumoral adipocytes (p<0.001, Figure 1E, 1F) and distant metastases (p<0.05; Figure 1G) were significantly more frequent in the high fat diet group, but organ-specific metastasis was not observed (Figure 1G). Despite the higher rates of primary tumor growth and distant metastasis in the high fat diet group, overall survival did not differ significantly in these two groups (Supplementry Figure 1G).

### Cancer cell colonies in the fat invasion model are scattered and surrounding fibrosis is increased

**Figure 2:**
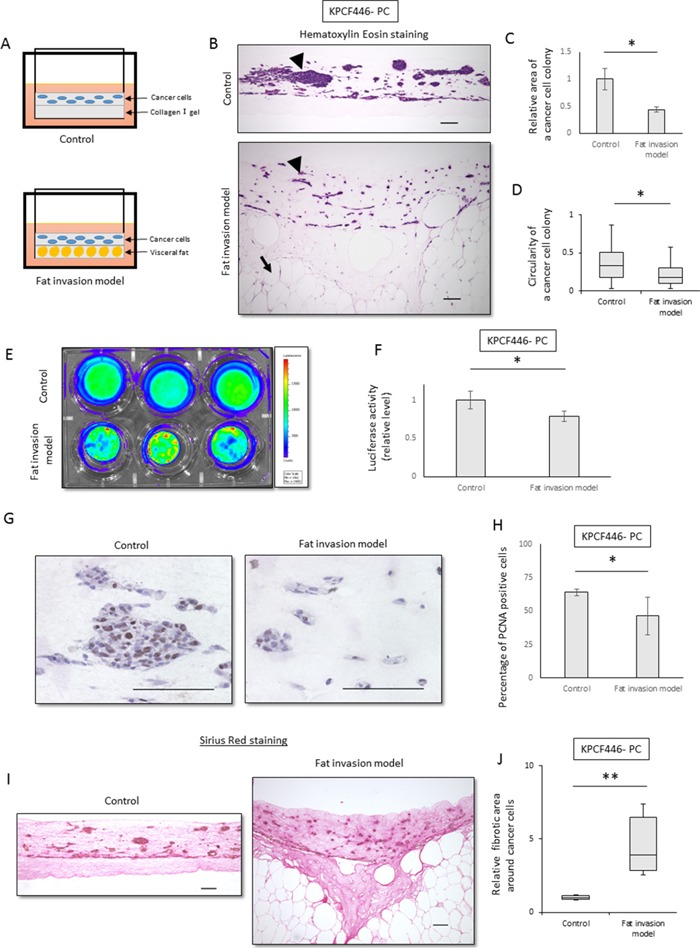
Cancer cell colonies in the fat invasion model are scattered and surrounding fibrosis was increased **(A)** Scheme of the fat invasion model. Minced murine visceral fat was embedded in the lower collagen I gel layer, and KPC tumor-derived cancer cells were embedded in the upper collagen I gel layer. In the control model, the upper layer was identical but the lower layer contained collagen I alone. **(B)** Representative histological images of the control and the fat invasion models stained with hematoxylin-eosin. Arrowheads indicate colonies of cancer cells (KPCF446-PC) in the upper layer, whereas arrows indicate adipocytes in the lower layer. Scale bar, 100 μm. **(C, D)** Effects of visceral fat on the morphology of cancer cell colonies (KPCF446-PC). **(C)** Colonies were smaller in the fat invasion than in the control model, as assessed by Image J software. Data are represented as mean ± standard error (SE). *p<0.05. **(D)** Cancer cell colonies were more elongated in the fat invasion than in the control model. The graph shows the circularity of each colony, as calculated by ImageJ. *p<0.05. **(E, F)** Effects of visceral fat on luciferase activity of cancer cells (KPCF446-PC). Luciferase activity was lower in the fat invasion than in the control model. Data are represented as mean ± standard deviation (SD). *p<0.05. **(G, H)** Effects of visceral fat on the numbers of PCNA-positive cells (KPCF446-PC). **(G)** Representative images of immunohistochemical assays for PCNA expression. Scale bar, 100 μm. **(H)** The PCNA positivity rate was lower in the fat invasion than in the control model. *p<0.05. **(I, J)** Effects of visceral fat on fibrotic areas around cancer cells (KPCF446-PC). **(I)** Representative images of Sirius red stained samples. Scale bar, 100 μm. **(J)** Fibrotic areas surrounding cancer cells were significantly greater in the fat invasion than in the control model. *p<0.05.

To analyze the mechanism underlying extra-pancreatic fat invasion, an *in vitro* fat invasion model, mimicking sites of peripancreatic fat invasion of pancreatic cancer, was established (Figure 2A, Supplementary Figure 2A). Visceral fat from a healthy mouse was minced into pieces and embedded in collagen I gel. The embedded fat maintained its histological appearance for 3 weeks when cultured in Dulbecco's modified Eagle's medium (DMEM) containing 10% fetal bovine serum (FBS) (Supplementary Figure 2B).

The fat invasion model consisted of two layers. The lower layer was composed of fat tissue and the upper layer of pancreatic cancer cells obtained from a pancreatic tumor of a KPC mouse (Supplementry Figure 2C), with both embedded in collagen I gel. As a control, we used a model containing the same upper layer, with the lower layer composed of collagen I gel alone (Figure 2A).

The areas of cancer cell colonies were significantly smaller (p<0.05, Figure 2B, 2C) and the cancer cells significantly more elongated (p<0.05, Figure 2B, 2D) in the fat invasion than in the control model. The proliferation of cancer cells was significantly lower in the fat invasion model, as shown by luciferase assays (p<0.05, Figure 2E, 2F) and PCNA immunohistochemistry (p<0.05, Figure 2G, 2H). Fibrosis around the tumor cells was significantly greater in the fat invasion than in the control model (p<0.05, Figure 2I, 2J, Supplementry Figure 2E, 2F), with the former being similar in histologic appearance to KPC mouse tumors and human PDACs.

### Effects of adipose tissue-derived conditioned medium on the migration, invasiveness, and gemcitabine resistance of pancreatic cancer cells

**Figure 3:**
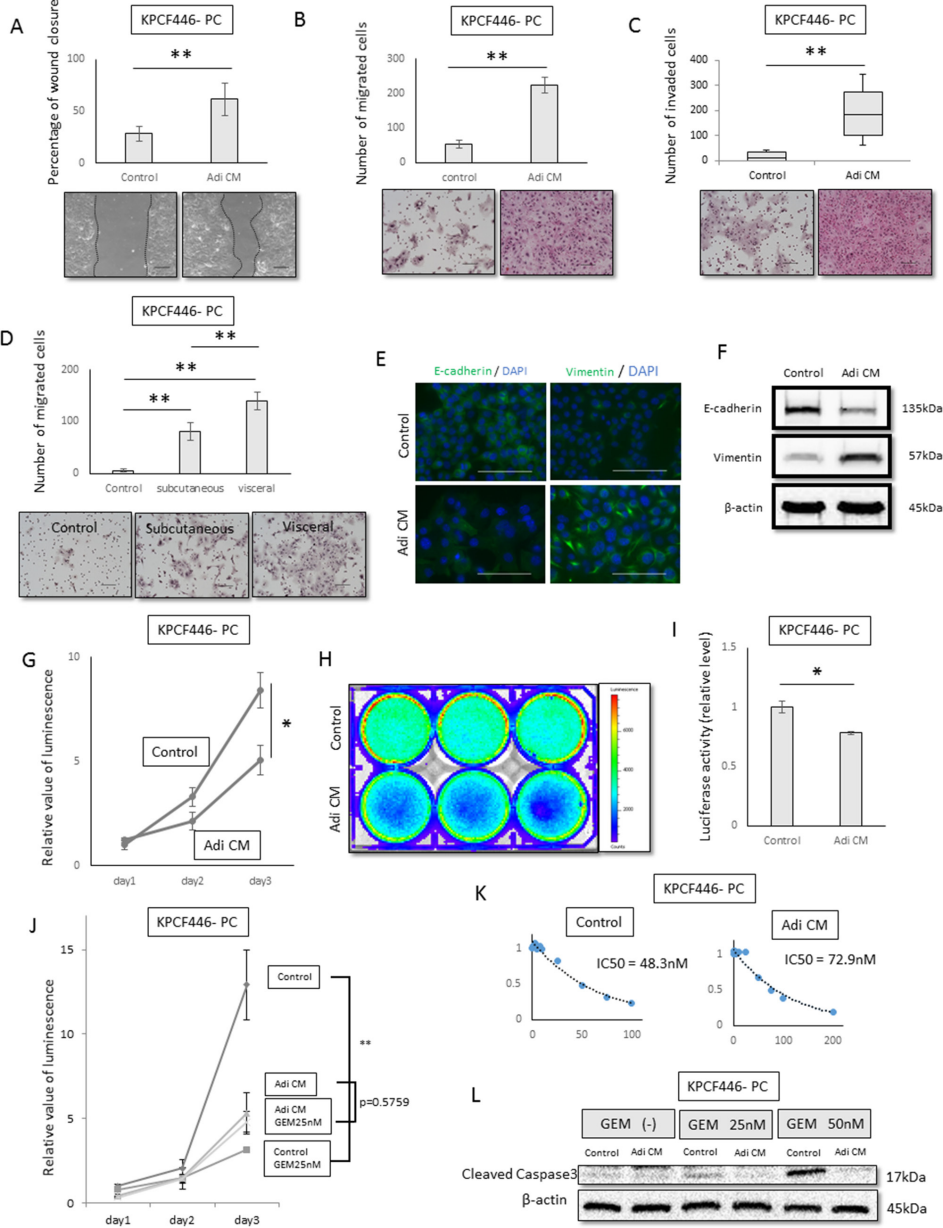
Effects of adipose tissue conditioned medium (Adi CM) on pancreatic cancer cell function *in vitro* **(A, B, C)** Wound healing **(A)** transwell migration **(B)** and Matrigel invasion **(C)** assays show that Adi CM significantly increased the migration and invasiveness of pancreatic cancer cells (KPCF446-PC). Scale bar, 100 μm. **p<0.001. **(D)** Relative effects of visceral and subcutaneous fat conditioned medium on migration ability of pancreatic cancer cells (KPCF446-PC). Visceral fat conditioned medium was significantly more effective. Scale bar, 100 μm. Data are represented as mean ± standard deviation (SD). **p<0.001. **(E, F)** Effects of Adi CM on E-cadherin and vimentin expression of pancreatic cancer cells (KPCF446-PC), as shown by immunofluorescence **(E)** and immunoblotting **(F)** Scale bars, 100 μm. **(G, H, I)** Effects of Adi CM on cell viability in conventional 2D culture and luciferase activity in collagen embedded 3D culture, showing Adi CM significantly reduced viability of pancreatic cancer cells (KPCF446-PC). *p<0.05. **(J, K, L)** Effects of Adi CM on gemcitabine sensitivity of pancreatic cancer cells (KPCF446-PC). **(J)** IC50 of gemcitabine, **(K)** gemcitabine inhibition of cancer cell proliferation **p<0.001, and **(L)** expression of cleaved caspase 3.

To assess the effects of adipose tissue on pancreatic cancer cells, these cells were incubated *in vitro* with adipose tissue-derived conditioned medium (Adi CM), which consisted of DMEM containing 10% FBS incubated for 24–48 h with adipose tissue embedded in collagen I gel. Adi CM significantly enhanced the motility of cancer cells in wound healing (p<0.001, Figure 3A, Supplementry Figure 3A, 3B) and transwell migration (p<0.001, Figure 3B, Supplementry Figure 3C, 3D) assays. To investigate the visceral fat-specific effects on cancer cells, visceral fat was compared with subcutaneous fat. The migration ability of cancer cells was significantly greater following incubation with visceral than with subcutaneous fat (p<0.001, Figure 3D, Supplementry Figure 3G, 3H). In addition, Adi CM enhanced the invasiveness of tumor cells in the matrigel invasion assay (p<0.001, Figure 3C, Supplementry Figure 3E, 3F). Adi CM also reduced E-cadherin expression and increased vimentin expression (Figure 3E, 3F, Supplementry Figure 3I), suggesting that Adi CM induced epithelial to mesenchymal transition (EMT).

Next, we investigated the effects of Adi CM on the proliferation of cancer cells. Both cell viability and luciferase assays showed that Adi CM reduced the proliferation of these cells in conventional 2D culture (Figure 3G, Supplementry Figure 3J, 3K) and when embedded in 3D collagen I (Figure 3H, 3I). To investigate the effects of Adi CM on the chemoresistance of cancer cells, these cells were treated with gemcitabine. The IC50 of these cells, which was 48.3 nM in control medium, increased to 72.9 nM in the presence of Adi CM (Figure 3J, Supplementry Figure 3L, 3M), indicating that Adi CM attenuated the effect of gemcitabine compared with control medium (Figure 3K). In addition, Adi CM reduced the expression of cleaved caspase 3, a marker of apoptosis (Figure 3L, Supplementry Figure 3N, 3O).

### Adipose tissue conditioned medium increases lipid droplets in pancreatic cancer cells

**Figure 4:**
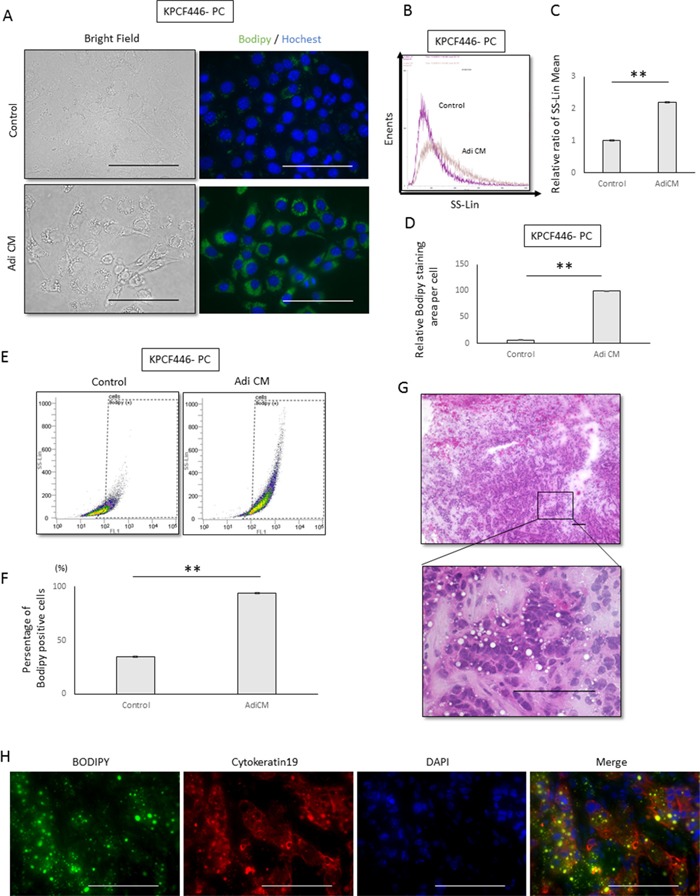
Effects of adipose tissue conditioned medium (Adi CM) on the morphology of pancreatic cancer cells **(A)** Representative bright field and Bodipy stained images of tumor cells (KPCF446-PC). Scale bars, 100 μm. **(B, C)** Adi CM increased cytoplasmic granules in cancer cells, as shown by side scatter analysis of flow cytometry results. **(B)** Representative side scatter histograms of cancer cells (KPCF446-PC) and **(C)** the bar chart obtained from three experiments. Data are represented as mean ± standard deviation (SD). **p<0.001. **(D)** Imaging analysis showing that Adi CM significantly increased Bodipy stained areas of cancer cells (KPCF446-PC). Data are represented as mean ± standard deviation (SD). **p<0.001. **(E, F)** Adi CM significantly increased the number of Bodipy positive cells, as shown by flow cytometry. **(E)** Representative dot plot graph of Bodipy positive cells and **(F)** a bar chart showing averages from three experiments. Data are represented as mean ± standard deviation (SD). **p<0.001. **G, H**. Representative images of a frozen section of a KPC tumor stained with **(G)** hematoxylin-eosin and **(H)** Bodipy, anti-cytokeratin 19, and DAPI. Scale bars, 100 μm.

We also tested the effects of Adi CM on cancer cell morphology. Flow cytometry side scatter analysis showed that culture with Adi CM increased the number of granular structures in the cytoplasm (Figure 4A, 4B, 4C). Bodipy staining showed that lipid droplets, which are located in granular structures, were increased after incubation with Adi CM (Figure 4A, 4D, Supplementry Figure 4A, 4B), and flow cytometry analysis of Bodipy-stained cells showed a significant increase in lipid positive cancer cells (Figure 4E, 4F, Supplementry Figure 4C, 4D). Increases in the number of lipid droplets in cancer cells were also observed in KPC pancreatic tumors *in vivo* (Figure 4G, 4H).

### Fatty acid uptake into cancer cells increased their migration and invasiveness

**Figure 5:**
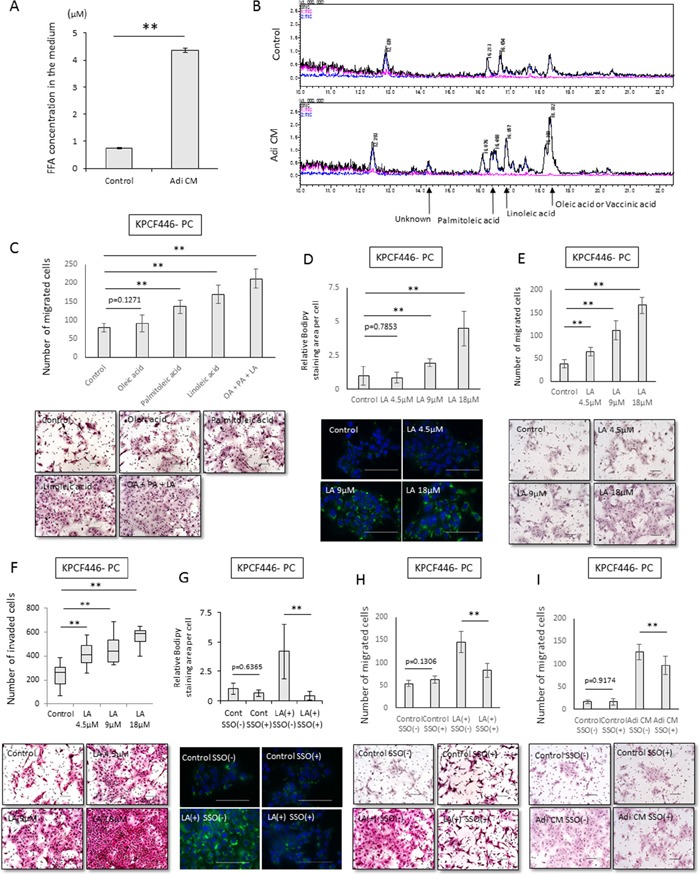
Effects of Adi CM on fatty acid incorporation into cancer cells and tumor cell migration **(A)** Measurement of fatty acids in Adi CM and control medium using Free Fatty Acid Quantification Kits. Data are represented as mean ± standard deviation (SD). **p<0.001. **(B)** Total ion chromatogram showing that concentrations of oleic, palmitoleic, and linoleic acids were higher in Adi CM than in control medium. **(C)** Effects of fatty acids on migration of cancer cells (KPCF446-PC). Scale bars, 100 μm. Data are represented as mean ± standard deviation (SD). **p<0.001. **(D, E, F)** Relationship between Bodipy stained areas of pancreatic cancer cells (KPCF446-PC) and the concentration of linoleic acid in the medium. **(D)** Linoleic acid dose-dependently increased cytoplasmic Bodipy-stained areas in cancer cells. Scale bars, 100 μm. Data are represented as mean ± standard deviation (SD). **p<0.001. **(E)** Transwell migration and **(F)** Matrigel invasion assays showing that migration and invasiveness increased in proportion to the concentration of linoleic acid in the medium. The incubation time for Matrigel invasion was 72 h. Scale bar, 100 μm. ** p<0.001. **(G)** Sulfo-N-succinimidyl oleate (SSO) inhibits fatty acid uptake into pancreatic cancer cells. (KPCF446-PC). Scale bars, 100 μm. Data are reported as mean ± standard deviation (SD). **p<0.001. **(H, I)** SSO inhibits linoleic acid- and Adi-CM-induced migration of tumor cells (KPCF446-PC). Scale bars, 100 μm. Data are shown as mean ± standard deviation (SD). **p<0.001.

We also analyzed the uptake of free fatty acids by cancer cells and the effects on malignancy. The concentration of free fatty acids was higher in Adi CM than in DMEM containing 10% FBS (Figure 5A), with total ion chromatography showing that the concentrations of oleic, linoleic and palmitoleic acids were higher in Adi CM than in normal medium (Figure 5B). Each of these three types of fatty acid enhanced the migration ability of cancer cells, with linoleic acid having the greatest effect (Figure 5C). Treatment with linoleic acid dose dependently increased the number of lipid droplets in cancer cells (Figure 5D), as well as their migration and invasiveness (Figure 5E, 5F, Supplementry Figure 4E–4L). In contrast, these fatty acids did not dose-dependently enhance the proliferation of cancer cells (Supplementry Figure 4M). Treatment of these cells with sulfo-N-succinyl oleate (SSO), an inhibitor of CD36 fatty acid translocase, reduced the number of lipid droplets in cancer cells, suggesting that the increase of lipid droplets following incubation with fatty acids may have been due to the direct uptake of fatty acids (Figure 5G). In addition, SSO inhibited the enhancement of cancer cell migration by linoleic acid and Adi CM (Figure 5H, 5I).

### Relationship between pancreatic cancer cells and lipolysis

**Figure 6:**
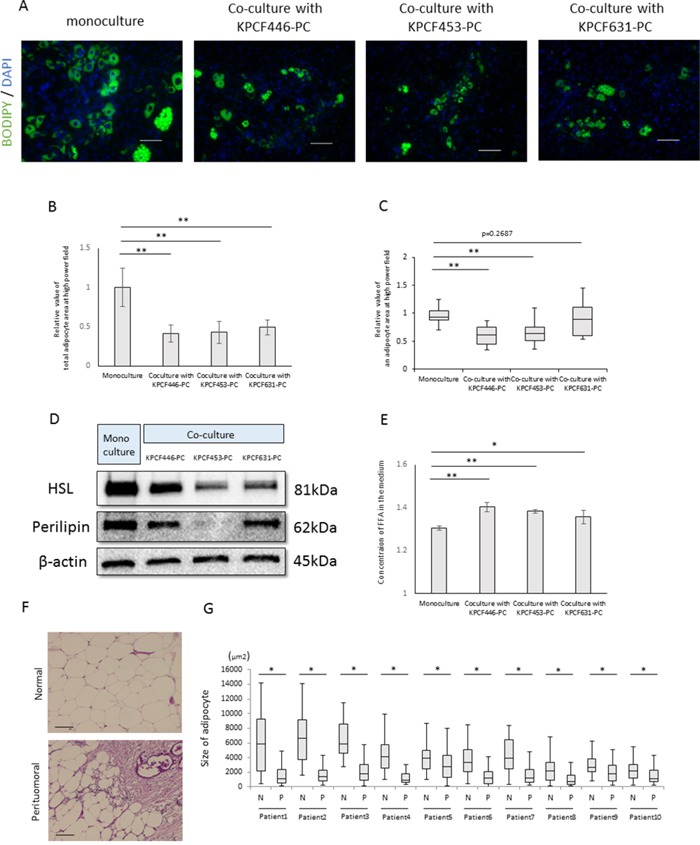
Effects of cancer cells on mature adipocyte size **(A)** Representative images of mature adipocytes mono-cultured or indirectly co-cultured with cancer cells stained with Bodipy and Hoechst reagent. Scale bars, 100 μm. **(B)** Relative total areas of adipocytes in a high power field. Data are reported as mean ± standard deviation (SD). **p<0.001. **(C)** Relative areas of single adipocytes in a high power field. **p<0.001. **(D)** Immunoblotting showing that expression of hormone sensitive lipase (HSL) and perilipin in mature adipocytes was lower in mature adipocytes cultured with than without cancer cells. **(E)** Measurement of fatty acid concentrations in the medium using Free Fatty Acid Quantification Kits. Data are reported as mean ± standard deviation (SD). *p<0.05, **p<0.001. **(F)** Representative images of a human PDAC and of peritumoral and normal adipocytes. Scale bars, 100 μm. **(G)** Peritumoral adipocytes were significantly smaller than normal adipocytes (n=10). *p<0.05.

To investigate the effects of factors released by cancer cells on adipocyte morphology, we established adipose tissue-derived stem cells, or DFAT cells, from murine visceral fat [23]. DFAT cells differentiated into mature adipocytes after culture in insulin-containing medium. Indirect culture of these mature adipocytes with cancer cells for 6 days reduced adipocyte size (Figure 6A, 6B, 6C) and expression of hormone sensitive lipase (HSL) and perilipin, both of which are adipocyte marker proteins (Figure 6D), while increasing the concentration of fatty acids in the medium (Figure 6E). Similarly, adipocytes at the invasive front of human PDACs were found to be significantly smaller than adipocytes located at sites distant from the tumor (Figure 6F, 6G). These results indicate that cancer cells induce lipolysis and promote the release of fatty acids from adipocytes.

## DISCUSSION

This study investigated the effects of visceral fat on the local invasiveness and metastasis of PDACs using a genetically engineered KPC (*Pdx1-Cre; LSL-Kras^G12D^; Trp53^R172H/+^*) mouse model, in which pancreatic epithelial cells express endogenous Kras and Trp53 mutations [24]. The clinical course and histological characteristics of this model resemble those of human PDAC, making this model superior to conventional xenograft models for studying pancreatic cancer. Although the KC (*Pdx1-Cre; LSL-Kras^G12D^*) mouse model also involves the induction of pancreatic intraepithelial neoplasia (PanIN) and invasive carcinoma, the incidence of the latter is lower than in KPC mice [25]. Feeding KC mice a high fat diet also increased the occurrence of PanIN and invasive carcinoma, suggesting that obesity was a risk factor for carcinogenesis [19–21]. The present study found that a high fat diet accelerated the growth of primary tumors and the frequency of distant metastases in KPC mice, in agreement with recent findings [26]. One of the primary histological features of pancreatic tumors is the increase in intratumoral adipocytes. Increased visceral fat may affect tumor growth at the invasive front, as well as enhancing distant metastases. In addition to increasing visceral fat, the high fat diet was found to induce diabetes mellitus [27], hyperglycemia, and arteriosclerosis. Thus, additional studies are needed to identify the factor(s) responsible for enhanced tumor growth in this mouse model.

Interactions between cancer cells and adipocytes have been found to promote the growth of pancreatic cancers. Adipocytes were shown to promote the proliferation of cancer cells via hepatocyte growth factor (HGF) signaling [28], and Wnt5a secreted by cancer cells was found to induce lipolysis [29]. Cancer cell-derived exosomes induce lipolysis in subcutaneous fat via a mechanism related to cachexia [30]. However, the 3T3-L1 and 3T3-F442A cells used in these studies were derived from mouse embryos, not from adult visceral fat. In focusing on local invasion, however, the present study used murine visceral fat, because adipocytes differ functionally depending on location [31]. An organotypic fat invasion model using cells embedded in collagen and cultured in three dimensions was therefore developed. Cancer cell migration was significantly greater when the cells were cultured in medium conditioned in visceral than in subcutaneous fat, in agreement with findings in ovarian cancer [14]. In addition, the organotypic fat invasion model revealed morphological changes in cancer cells and fibrotic changes around these cells reflective of pancreatic tumor histology. Culture of organotypic fat embedded in collagen is a simple but suitable method of assessing the interactions between cancer cells and adipocytes *in vitro*, as well as being applicable to other cancers.

Adipocytes store triglycerides in their cytoplasm, releasing glycerol and fatty acids in response to extra-cellular stimuli [32]. The role of fatty acids in pancreatic cancer, however, remains poorly understood. Fatty acids were found to alter the proliferation of pancreatic cancer cells *in vitro*, and n-6 polyunsaturated fatty acids were shown to promote the liver metastasis of a xenograft tumor model *in vivo* [33]. In the present study, we found that culture of cancer cells in adipose tissue conditioned medium increased the number of lipid droplets per cell, accompanied by increased invasiveness, reduced proliferation and enhanced chemoresistance *in vitro*. In a mouse melanoma model, sFRP2, a Wnt agonist, secreted by aged fibroblasts, was found to enhance the invasiveness and reduce the proliferation of cancer cells, a phenomenon called phenotype switching [34]. In pancreatic and breast cancer models, EMT has been associated with chemoresistance [35, 36]. Taken together, our results indicate that adipocyte secreted factor induced EMT in cancer cells, accompanied by phenotype switching and enhanced resistance to gemcitabine. Moreover, we found that fatty acids are important in interactions between cancer cells and adipocytes. To our knowledge, this is the first study to show that fatty acids promote the migration of pancreatic cancer cells *in vitro*, findings consistent with those in breast cancer [37, 38]. Adipocytes, however, not only store lipids but act as endocrine cells, releasing many cytokines and adipokines [39, 40]. These factors may influence the proliferation of cancer cells, as shown by the ability of Adi CM, but not fatty acids, to dose-dependently reduce the proliferation of cancer cells. Additional studies are needed to determine the molecular mechanisms underlying the interactions between cancer cells and adipocytes.

A new therapeutic strategy in cancer may be the targeting of stromal elements. Because the hedgehog inhibitor IPI-926 reduced extracellular matrices and increased the delivery of gemcitabine in KPC mice [4], targeting pancreatic stellate cells with this agent was expected to improve clinical outcomes. Clinically, however, IPI-926 had no significant benefits [41]. Moreover, ablation of stromal elements has been reported to increase the numbers of undifferentiated cancer cells, resulting in a poorer prognosis [41]. These findings indicate that stromal interactions are regulated by highly complex systems and that there is a need for further investigations of stromal biology in PDAC. This study focused on cancer-adipocyte interactions, which may occur at the invasive front of PDACs. We found that inhibition of fatty acid uptake reduced the migration of cancer cells, suggesting a novel future therapeutic target. However, additional studies on the expression of the fatty acid transporter protein (FATP) CD36 in pancreatic cancer cells are needed.

In conclusion, we found that pancreatic cancer cells induced lipolytic and fibrotic changes in the peripancreatic adipose environment. The interactions between adipocytes and cancer cells led to the release of fatty acids from the former. Increased fatty acid uptake by cancer cells enhanced cell invasiveness *in vitro* and both primary tumor growth and distant metastases *in vivo*. These findings suggest that inhibiting fatty acid uptake by pancreatic cancer cells may be a new therapeutic target to regulate their local invasiveness.

## MATERIALS AND METHODS

### Patients and pancreatic tissue

Tissue samples were obtained from patients who underwent pancreatic resection for pancreatic cancer at Kyushu University. The study was approved by the Ethics Committee of Kyushu University and was conducted according to the Ethical Guidelines for Human Genome/Gene Research enacted by the Japanese Government and the Helsinki Declaration. Ten samples with peripancreatic fat invasion were randomly selected. The sizes of peritumoral and normal adipocyte were measured using a Keyence Microscope and its exclusive analysis software (BZ-9000; Keyence, Japan). Normal adipocytes were defined as those located at a distant site from the tumor without inflammation or fibrosis.

### Transgenic mice and high fat diet

The *Pdx1-Cre; LSL-Kras^G12D^; Trp53^R172H/+^* mice have been previously characterized and genotyped [24]. These mice were randomly divided into two groups, with one group fed a general diet (CE-2, CLEA Japan, Inc.) and the other fed a high fat diet (HFD32, CLEA Japan, Inc.); in the latter, 56.7% of energy was derived from fat. The body weight of each mouse was measured weekly, and the presence of a pancreatic tumor was determined by palpation and MRI imaging. Mice were sacrificed when they became weak, lost a drastic amount of weight, or displayed obvious abdominal distention. Their pancreases, livers, lungs, and disseminated nodules were removed and fixed in 10% formalin (133-10311, Wako, Japan) for histological analysis.

C57BL/6NJcl mice were fed the high fat diet and their visceral fat was used for primary culture or the organotypic model. All animal experiments were approved by the Ethics Committee of Kyushu University.

### Cell isolation and culture conditions

Pancreatic cancer cells from primary tumors in KPC mice were established using an outgrowth method [42], and isolated cancer cell lines were maintained as described [43]. Cell lines were authenticated as positive for the epithelial cell markers cytokeratin 19 and E-cadherin and to be tumorigenic *in vivo*. Adipose tissue derived stem cells (DFAT cells) were also established from murine visceral fat as described [23]. These cells were authenticated by their ability to undergo adipogenic differentiation in preadipocyte differentiation medium (C-39425, Lonza, Basel, Switzerland). Differentiated mature adipocytes were maintained in DMEM (Sigma Chemical Co., St. Louis, MO, USA) containing 100 nM insulin (099-06473, Wako), 10% fetal bovine serum (FBS), 100 μg/ml streptomycin, and 100 units/ml penicillin. Oleic acid (O1257), palmitoleic acid (P9417), and linoleic acid (L5900) were obtained from Sigma-Aldrich and dissolved in DMEM containing 1% FBS to the desired concentration. A 200 μM solution of sulfosuccinimidyl Oleate (SSO; 11211, Cayman, Ann Arbor, MI, USA) was added to the medium to inhibit lipid uptake.

### Immunohistochemistry

Immunohistochemical staining was performed as described [44]. Briefly, formalin-fixed, paraffin-embedded tissue samples were sliced and deparaffinized with xylene and ethanol. Endogenous peroxidase activity was blocked by incubation with 0.3% hydrogen peroxidase in methanol. Antigen retrieval was performed by immersion of tissue samples in citrate buffer and boiling in a microwave oven. The sections were incubated at 4°C with primary antibody against anti-PCNA (ab2426, Abcam, Cambridge, UK; 1:500), followed by incubation with secondary antibody (EnVision System; K4002, Dako, Troy, MI, USA) at room temperature for 40 min and visualization with 3,3′-diaminobenzidine.

### Organotypic peripancreatic fat invasion model

The organotypic model mimicking peripancreatic fat invasion of PDAC was a modification of a previous method [45, 46]. Briefly, the lower layer consisted of murine visceral fat minced into pieces and embedded in collagen I gel (#354235, Corning, NY, USA), and the upper layer consisted of pancreatic cancer cells from a KPC mouse. The final concentration of collagen I gel was adjusted to 2 mg/ml with PBS, and one-tenth the volume of 10× DMEM and Reconstruction Buffer (Nitta Gelatin, Japan) was added. The tissue samples were cultured in DMEM containing 10% FBS for 2 weeks and fixed in 4% paraformaldehyde (163-20145, Wako). Fixed samples were embedded in paraffin, sectioned, mounted onto glass slides, and stained with hematoxylin-eosin. Shapes of the cancer cell colonies were evaluated using Image J software [47]. Fibrosis was evaluated by staining with Masson Trichrome and Sirius Red.

### Collection of adipose tissue-conditioned medium

For culturing adipose tissue, cell culture inserts of pore size 3 μm (#353091, BD Falcon, Franklin Lakes, NJ, USA) were inserted into 6-well plates. About 1g of minced murine visceral fat embedded in collagen I gel (#354235, Corning), as described above, was incubated in 3ml of DMEM containing 10% FBS. The medium was collected 24–48 h later and centrifuged at 1500 rpm for 5 min. Supernatants were collected and stored at 4°C until used. Fresh DMEM containing 10% FBS was used as control.

### Wound healing, transwell migration, and matrigel invasion assays

Wound healing assays were performed using culture inserts (80209, Ibidi, Martinsried, Germany), as previously described [48, 49]. Briefly, 70 μl of 5 × 10^5^ cells/ml were seeded into each well and incubated overnight at 37°C. After allowing the cells to attach to the dish, the culture inserts were removed carefully, and control medium or adipose tissue conditioned medium was added. Time lapse imaging was performed for 6 h using a fluorescent microscope (BZ-9000; Keyence, Japan) and the percent closed area calculated by Image J software.

The transwell migration and matrigel invasion assays were performed as described [50, 51]. For migration assays, cell culture inserts of pore size 8 μm (#353097, BD Falcon, Franklin Lakes, NJ, USA) were inserted into 24-well plates. A 750 μl aliquot of medium was added to each lower chamber, and 5 × 10^4^ cancer cells resuspended in 250 μl were added to each upper chamber. The plates were incubated at 37°C for 24 h, except when comparing media conditioned with visceral and subcutaneous fat, when the incubation time was set at 16 h. The membranes were subsequently fixed with 70% ethanol and stained with hematoxylin-eosin.

For matrigel invasion assays, the membranes were coated with 20 μg/well of Matrigel (356234, Corning) and incubated for 48 h, except for the experiment using linoleic acid, when the incubation time was set at 72 h. The numbers of migrating and invading cells were counted in five random fields at 200× magnification using Image J software. The results were expressed as the mean number of invading cells. Each experiment was carried out in triplicate wells, and independent experiments were repeated three or more times.

### Immunoblotting

Protein was extracted from KPC tumor-derived cancer cells and mature adipocytes using PRO-PREP (17081, iNtRON biotechnology, Korea), according to the manufacturer's instructions, and stored at −80°C until used. Aliquots containing 20 μg of each sample were loaded onto Mini-PROTEAN TGX Precast Gels (#4561086, Bio-Rad Laboratories, Hercules, CA, USA) and electrophoresed at 200 V for about 30 min. Proteins were electrophoretically transferred to Turbo Mini PVDF membranes (#1704156, Bio-Rad Laboratories) using a Trans-Blot Turbo Transfer Starter System (Bio-Rad Laboratories). After blocking with skim milk, the membranes were incubated at 4°C overnight with 1:1000 dilutions of primary antibodies against E-cadherin (#3195), vimentin (#5741S), Cleaved caspase3 (#9661S), HSL (#4107), perilipin (#9349), and β-actin (#4970S), all obtained from Cell Signaling Technology (Danvers, MA, USA). After washing, the membranes were incubated with secondary antibody (#7074S, Cell Signaling; 1:5000) at room temperature for 1 h and with Clarity Western ECL Substrate (Bio-Rad Laboratories). The immunoblots were assessed using a ChemiDoc XRS enhanced chemiluminescence system (Bio-Rad Laboratories).

### Immunocytochemistry

Immunocytochemistry was performed as described [52]. Briefly, KPC tumor-derived cancer cells were fixed in 4% paraformaldehyde (163-20145, Wako) for 5 min at room temperature. After blocking with 3% BSA for 30 min, the cells were incubated at room temperature for 2 h with 1:100 dilutions of primary antibodies against E-cadherin (#3195), and vimentin (#5741S) from Cell Signaling Technology, and antibody to cytokeratin 19 (ab133496, Abcam). The cells were washed and incubated for 1 h at room temperature with Alexa Fluor secondary antibodies (A-11034, Thermo Fisher, Waltham, MA, USA ; 1:200).

### Cell proliferation assay

To evaluate cell proliferation in conventional 2D cultures, cell viability assays were performed. KPC tumor-derived cancer cells (1 × 10^3^ cells/well) were seeded in 96-well plates. After confirmation of cellular adhesion to the plates, the medium was replaced with fresh DMEM containing 10% FBS or adipose tissue-conditioned medium. After culture for 24–72 hours, cell proliferation was measured using Cell Titer-Glo Luminescent Cell Viability Assay Kit (G7570, Promega, Fitchburg, WI, USA), according to the manufacturer's instructions.

Cell proliferation in cultures of embedded 3D collagen I gel were evaluated by *in vitro* luciferase assays. Luciferase expressing cancer cells were prepared using lentiviral particles (LVP326, GenTarget Inc., San Diego, CA, USA), with 1 × 10^6^ cells embedded in collagen I gel in 6-well plates, followed by culture in standard medium or adipose tissue-conditioned medium for 7 days. After administration of 150 μg/mL D-luciferin (LK10000, OZBIOSCIENCE, Marseille, France), the plate was imaged using the IVIS 100 system.

### Cytoplasmic lipid droplet staining

Cells were washed with PBS and fixed with 10% formalin (133-10311, Wako) for 10 min. After three washes with PBS, the cells were incubated in the dark with 1 μM of Bodipy 493/503 (D-3922, Invitrogen, Waltham, MA, USA) and Hoechst 33342 (346-07951, Wako; 1:100) at room temperature for 30 min. The cells were subsequently visualized by a fluorescent microscope (BZ-9000; Keyence) for fluorescence microphotography.

### Flow cytometry

Cells cultured in control or adipose tissue-conditioned medium for 24 h were trypsinized and resuspended in PBS containing 2% FBS. Aliquots of 1 × 10^6^ cells were fixed in 4% paraformaldehyde (163-20145, Wako) for 5 min at room temperature, washed three times with PBS, and incubated in the dark with 10 nM Bodipy 493/503 (D-3922, Invitrogen) for 3min at room temperature. After two additional washes with PBS, the cells were analyzed by flow cytometry (EC800, Sony, Japan).

### Measurement of free fatty acids in the medium

Total concentrations of fatty acids in the medium were measured using a Free Fatty Acid Quantification Kit (AB65341, Abcam), according to the manufacturer's instructions. TIC chromatography was performed as described [53]. Briefly, samples were dissolved in chloroform, vortexed, and centrifuged at 15000 rpm for 5 min. The lower phase of each sample was collected and dried overnight. Fatty acid concentrations were measured by high performance liquid chromatography-tandem mass spectrometry.

### Statistical analysis

Data are reported as mean ± standard deviation (SD) and compared by Student's t tests, with a P value < 0.05 considered statistically significant. All *in vitro* experiments were repeated three times. The Kaplan-Meier method was used to analyze survival, with curves compared using the log-rank test. All statistical analyses were performed using JMP Pro 11 software (SAS Institute, Cary, NC, USA).

## SUPPLEMENTARY MATERIALS FIGURES


