# Supplementary material for: Extra-pancreatic invasion induces lipolytic and fibrotic changes in the adipose microenvironment, with released fatty acids enhancing the invasiveness of pancreatic cancer cells
Source: Oncotarget. 2017 Feb 17;8(11):18280–95. doi: 10.18632/oncotarget.15430 (PMC5392327; doi:10.18632/oncotarget.15430)
Supplement: Supplementary file 1 [file oncotarget-08-18280-s001.pdf]

Extra-pancreatic invasion induces lipolytic and fibrotic changes in the adipose microenvironment, with released fatty acids enhancing the invasiveness of pancreatic cancer cells

SUPPLEMENTARY FIGURES

A

	Crude moisture (%)	Crude protein (%)	Crude fat (%)	Crude fiber (%)	Crude Ash (%)	Energy (kcal/100g)
Control (CE-2, CLEA Japan)	8.89	24.88	5.03	4.63	6.79	343.9
High fat diet (HFD32, CLEA Japan)	6.2	25.5	32.0	2.9	4.0	507.6

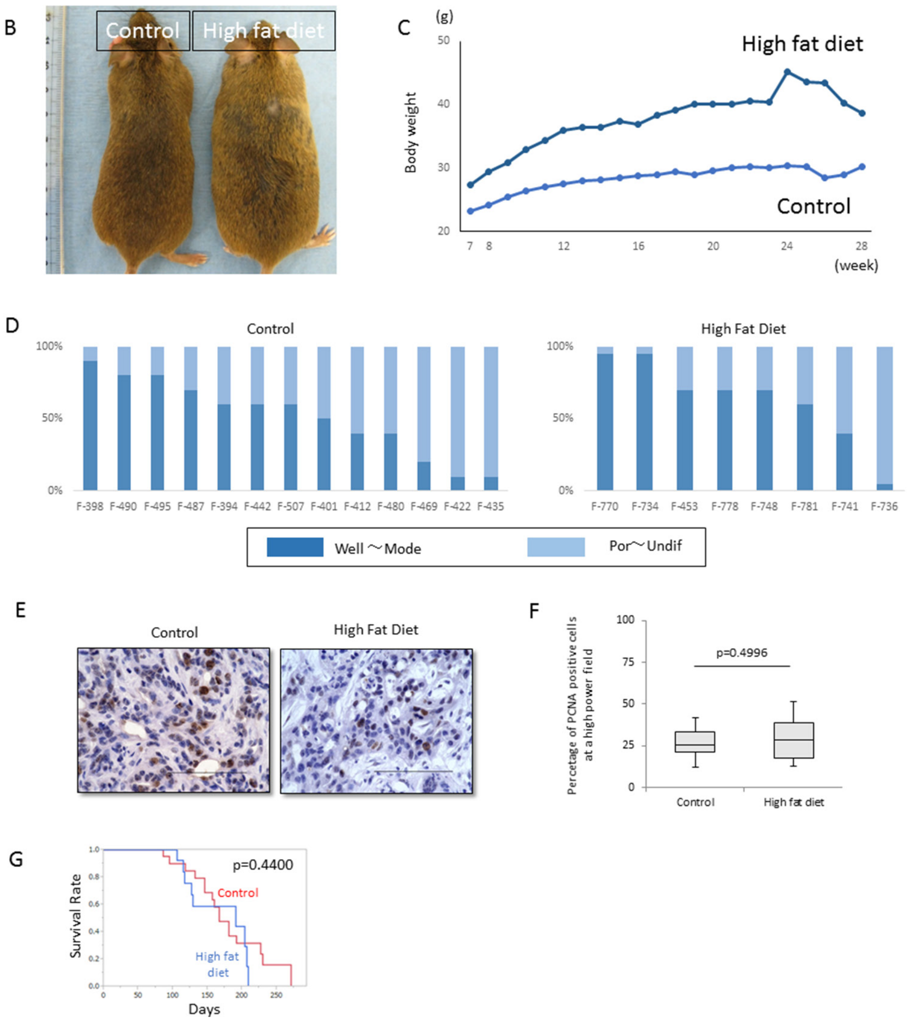

Supplementary Figure 1: (A) Details of the composition of control (CE2, CLEA Japan) and high fat (HFD32, CLEA Japan) diets. (B) Representative photograph of KPC mice fed the control and high fat diets. **(C)** Line chart showing serial changes in mean body weight of KPC mice. **(D)** Similar degree of differentiation of KPC tumors in mice fed a normal (n=13) and high fat (n=8) diet. **(E, F)** Effects of high fat diet on PCNA expression. **(E)** Representative image of PCNA immunohistochemistry. Scale bar, 100 µm. **(F)** The number of PCNA-positive cells was similar in KPC tumors of mice fed a normal (n=13) and a high fat (n=8) diet. **(G)** Kaplan-Meier curves of overall survival in mice fed a high fat (n=9) and a normal (n=16) diet, with comparisons by the log-rank test.

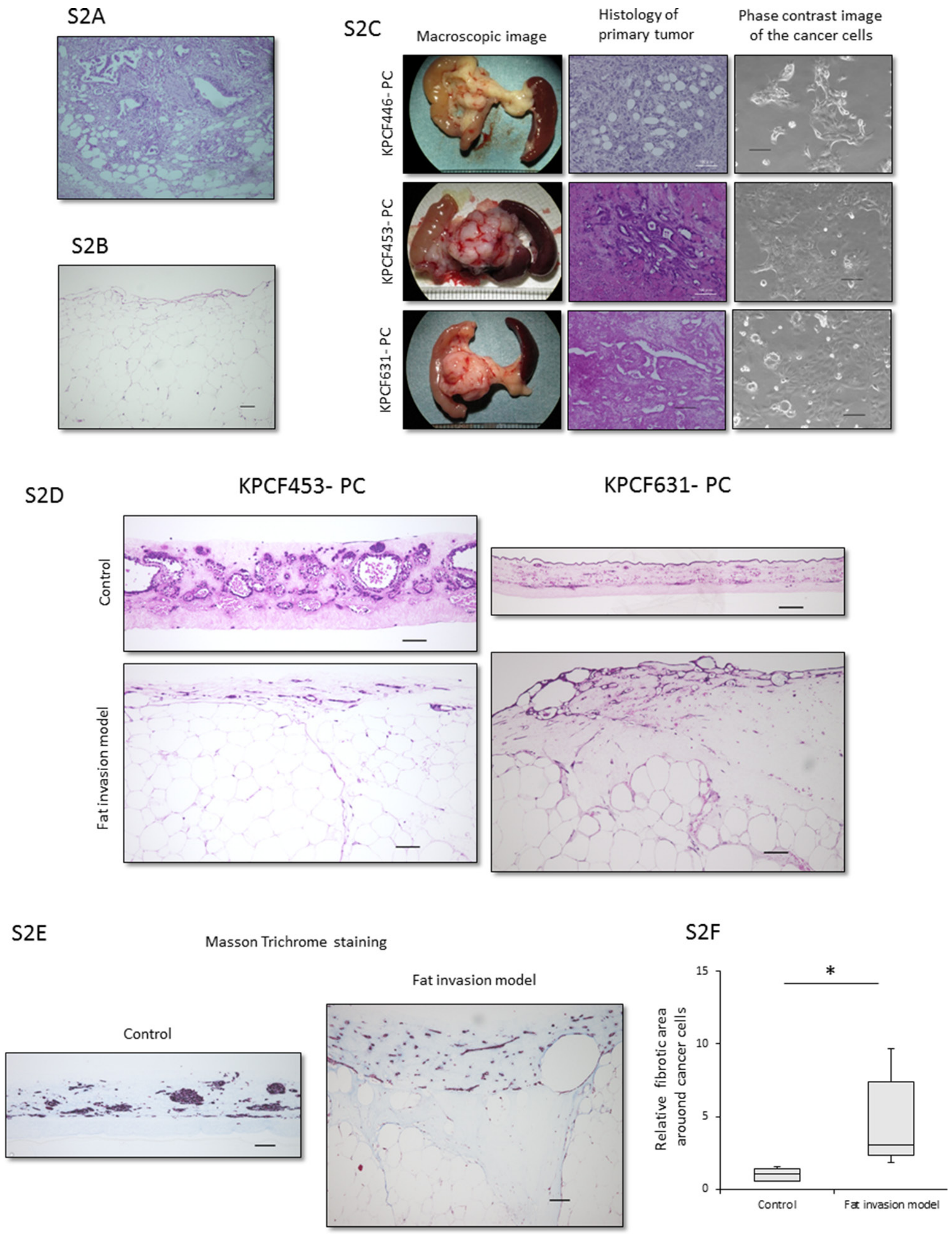

Supplementary Figure 2: (A) Representative histological image of a human PDAC at the peripancreatic fat invasion site. Original magnification ×100. **(B)** Representative histological image of cultured adipose tissue embedded in collagen I gel stained with hematoxylin-eosin. Scale bar, 100 µm. **(C)** Representative macroscopic and histologic images of KPC primary tumors and phase contrast image of established cancer cells. Scale bars, 100 µm. **(D)** Representative histological images of the control and organotypic fat invasion models stained with hematoxylin-eosin (KPCF453-PC, KPCF631-PC). Scale bars, 100 µm. **(E)** Representative histological images of the control and organotypic fat invasion models stained with Masson trichrome. Scale bars, 100 µm. **(F)** Fibrotic areas around cancer cells (KPCF446-PC) were greater in the fat invasion than in the control model. *p<0.05.

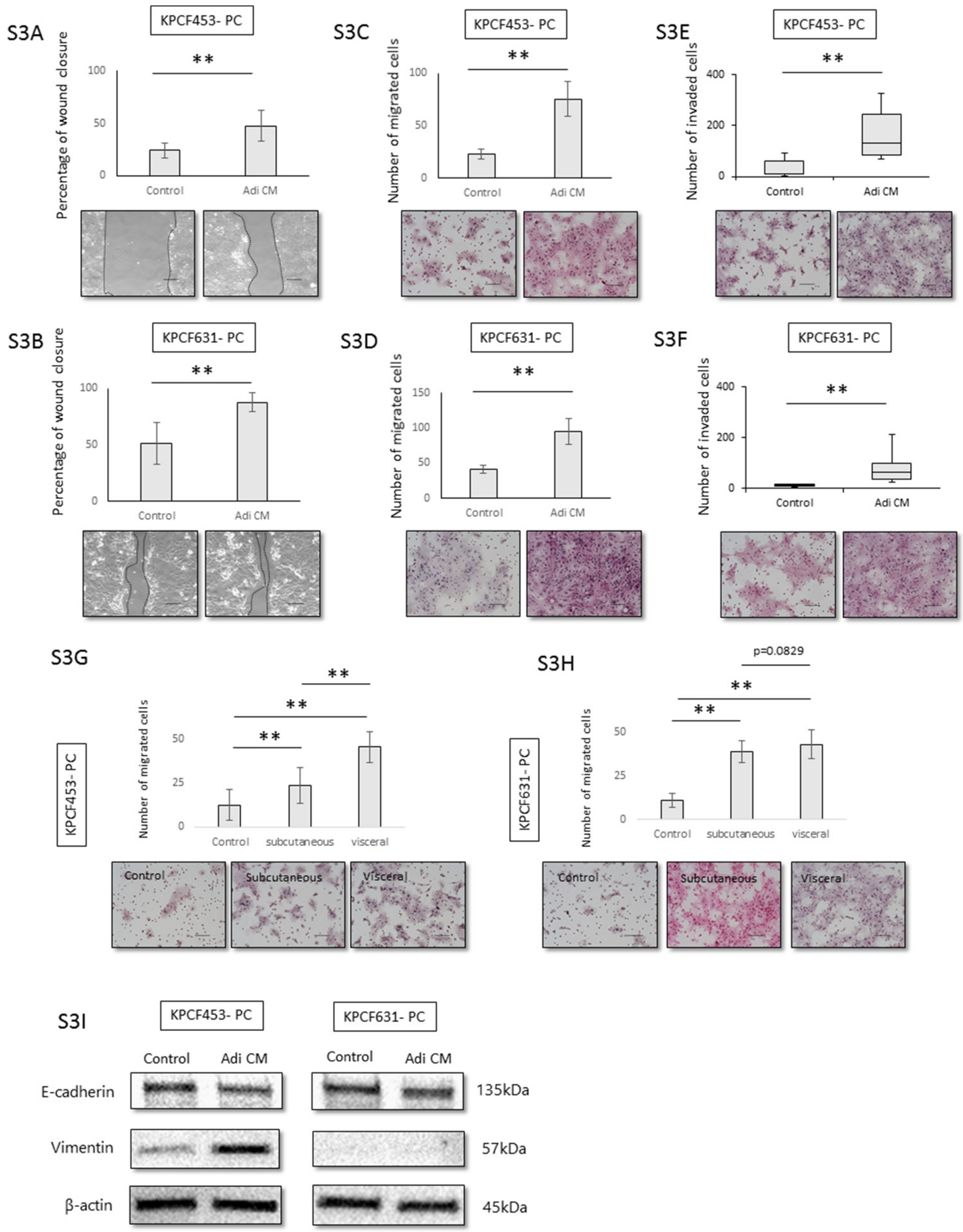

Supplementary Figure 3: Effects of Adi CM on tumor cell properties. **(A–F)** Wound healing **(A,B)** transwell migration **(C,D)** and Matrigel invasion **(E,F)** assays, showing that Adi CM significantly increased the migration and invasiveness of tumor cells (KPCF453-PC, KPCF631-PC). Scale bars, 100 µm. **p<0.001. **(G, H)** Relative effects of visceral and subcutaneous fat conditioned medium on cell migration (KPCF453-PC, KPCF631-PC). Scale bars, 100 µm. Data are reported as mean ± standard deviation (SD). **p<0.001. **(I)** Effects of Adi CM on E-cadherin and vimentin expression, as shown by immunoblotting (KPCF453-PC, KPCF631-PC).

(*Continued*)

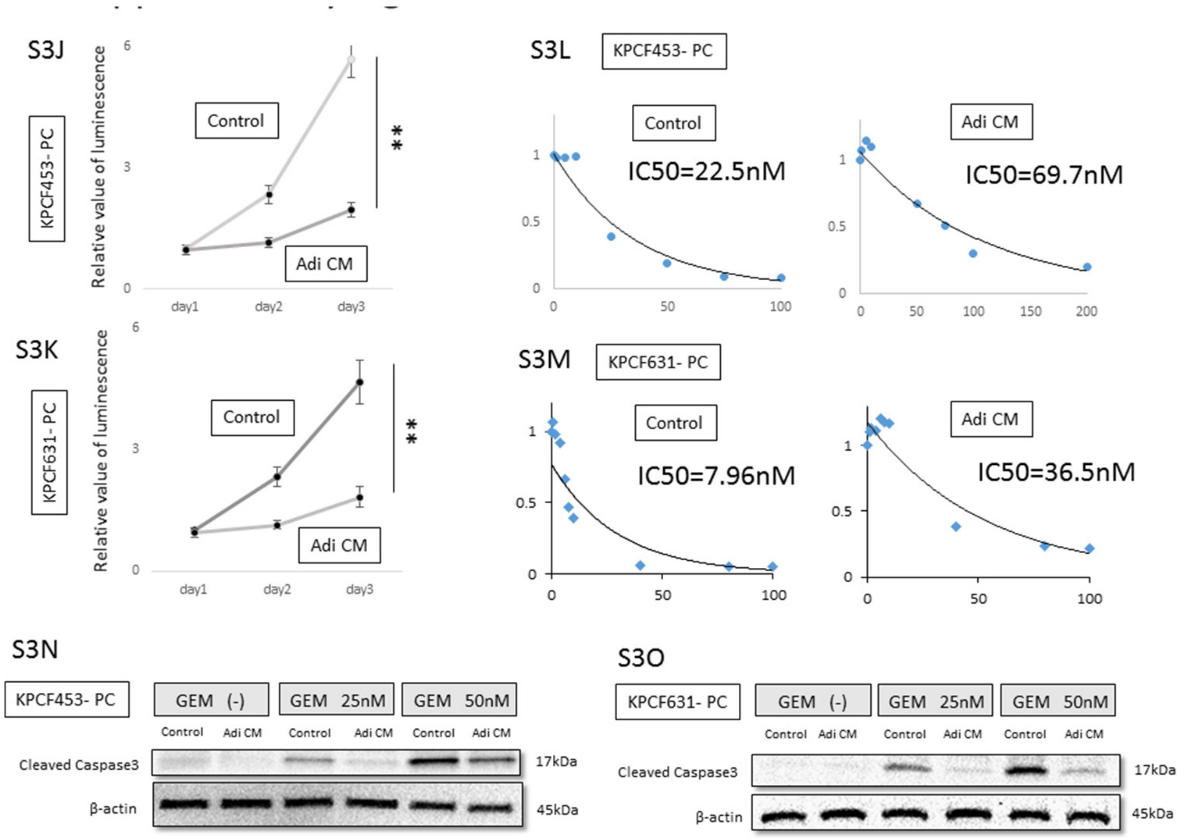

Supplementary Figure 3 (*Continued*): (J, K) Effects of Adi CM on cell viability in conventional 2D cultures (KPCF453-PC, KPCF631-PC). **$p<0.001$. **(L, M)** Effects of Adi CM on IC50 of gemcitabine (KPCF453-PC, KPCF631-PC). **(N, O)** Effects of Adi CM on cleaved caspase 3 expression following treatment with gemcitabine (KPCF453-PC, KPCF631-PC).

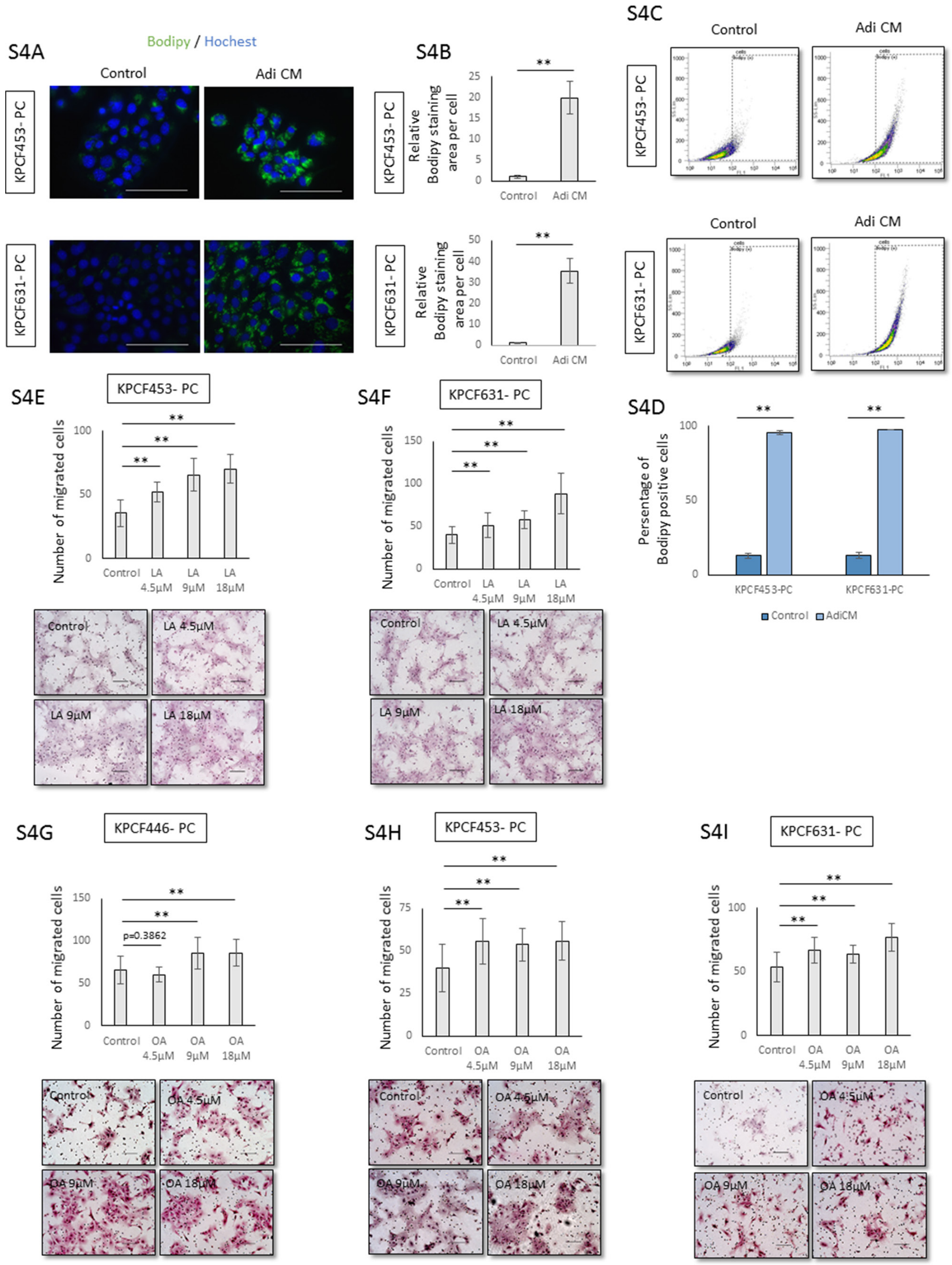

Supplementary Figure 4: (A) Representative bright field and Bodipy stained images (KPCF453-PC, KPCF631-PC). Scale bars, 100 μm. **(B)** Imaging analysis showing that Adi CM significantly increased Bodipy stained areas in cells (KPCF453-PC, KPCF631-PC). Data are reported as mean ± standard deviation (SD). **p<0.001. **(C, D)** Flow cytometry showing that Adi CM increased the percentage of Bodipy positive cells (KPCF453-PC, KPCF631-PC). **(C)** Representative dot plot graph of Bodipy positive cells and **(D)** the bar chart obtained from three experiments. Data are reported as mean ± standard deviation (SD). **p<0.001.

(*Continued*)

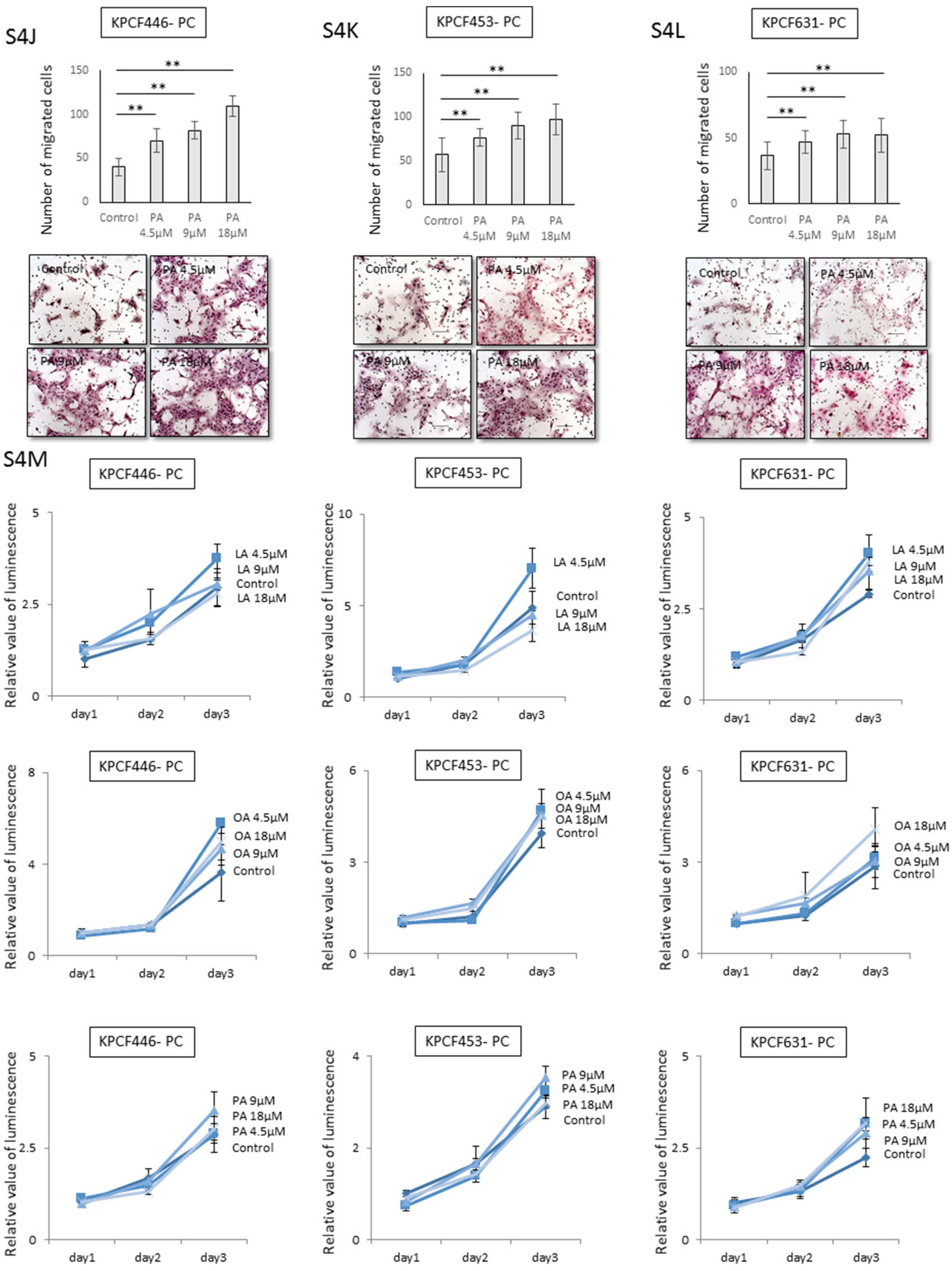

Supplementary Figure 4 (*Continued*): (E–L) Transwell migration assays showing that (E, F) linoleic acid, (G, H, I) oleic acid, and (J, K, L) palmitoleic acid in the medium dose-dependently enhanced tumor cell migration. Data are reported as mean ± standard deviation (SD) Scale bars, 100 μm. **p<0.001. **(M)** Effects of fatty acids on cancer cell proliferation.
